# Changes in older persons’ lifestyle and perceived health over time and during the COVID-19 pandemic: findings from the extended follow-up of the FINGER randomized controlled trial from 2009 to 2020

**DOI:** 10.1186/s12877-025-05979-6

**Published:** 2025-05-03

**Authors:** Jenni Lehtisalo, Maria Sääskilahti, Tommi Härkänen, Jenni Kulmala, Katri Hemiö, Sini Siltanen, Zhou Zhi, Francesca Mangialasche, Tiina Laatikainen, Timo Strandberg, Riitta Antikainen, Jaakko Tuomilehto, Hilkka Soininen, Miia Kivipelto, Tiia Ngandu

**Affiliations:** 1 Department of Public Health, Lifestyles and Living Environments, Finnish Institute for Health and Welfare, Helsinki, 00271 Finland; 2 Department of Clinical Medicine/Neurology, University of Eastern Finland, Kuopio, 70211 Finland; 3 Division of Clinical Geriatrics, Center for Alzheimer Research, Department of Neurobiology, Care Sciences and Society, Karolinska Institutet, Solna, Stockholm, 171 64 Sweden; 4 Faculty of Social Sciences (Health Sciences) and Gerontology Research Center (GEREC), Tampere, Finland; 5 FINGERS Brain Health Institute, Stockholm, Sweden; 6 Theme Inflammation and Aging, Medical Unit Aging, Karolinska University Hospital, Stockholm, Sweden; 7 Present Address: Institute of Public Health and Clinical Nutrition, University of Eastern Finland, Kuopio, Finland; 8 University of Helsinki and Helsinki University Hospital, Helsinki, Finland; 9 Research Unit of Population Health/Geriatrics, University of Oulu, Oulu, FI-90014 Finland; 10 Center for Geriatrics and General Medicine, Oulu University Hospital, Pohde Wellbeing Services County of North Ostrobothnia, Oulu, Finland; 11 Medical Research Center Oulu, Oulu University Hospital, Oulu, Finland; 12 South Ostrobothnia Central Hospital, Seinäjoki, Finland; 13 Department of Public Health, University of Helsinki, Helsinki, Finland; 14 Diabetes Research Group, King Abdulaziz University, Jeddah, Saudi Arabia; 15 Department of Neurology, Kuopio University Hospital, Kuopio, Finland; 16 The Ageing Epidemiology Research Unit, School of Public Health, Imperial College London, London, UK

**Keywords:** Lifestyle, Intervention, Change, Covid-19 pandemic, Follow-up

## Abstract

**Background:**

Multidomain lifestyle trials have been shown to be effective in changing people’s behaviour during the intervention, but less is known about long-term effects of such interventions. The aim of this study was to investigate how self-reported lifestyle and self-evaluated health changed over a 10-year period in older adults participating in the FINGER randomised controlled trial. Effects of the initial lifestyle intervention and the COVID-19 pandemic on these behaviour changes were evaluated.

**Methods:**

A two-year multicentre FINGER trial recruited community-dwelling people aged 60-77 years at risk of cognitive impairment (*n*=1259). Participants were randomised to a multidomain lifestyle intervention or regular health advice (control). They underwent study visits annually during the original trial period (at baseline, one, and two years) and twice during the follow-up (five and seven years), and responded to a survey during the COVID-19 pandemic at approximately 10 years. Generalised estimating equations (GEE) and linear mixed-effects regression model were used to analyse physical, cognitive, and social activity, food consumption, smoking, alcohol consumption, and self-evaluated health.

**Results:**

People in the intervention group were better able to maintain their level of physical activity up to the five-year follow-up. The intervention group also improved their diet quality: difference in fish consumption was maintained up to the seventh year, and consumption of vegetables and fruits increased during the active intervention. Cognitive and social activities increased and self-evaluated health and memory improved during the active period, but decreased thereafter, without a group difference. During the COVID-19 pandemic, physical and cognitive activities increased.

**Conclusion:**

Multidomain lifestyle intervention was beneficial for improving physical activity and healthy food choices in older people both in the short and long term, but had no effect on other activities, smoking, alcohol use, or self-evaluated health. Increased physical activity was the most evident pandemic-associated change in older adults’ lifestyle.

**Trial registration:**

ClinicalTrials.gov, NCT01041989. Registered 04/01/2010 – Retrospectively registered, https://clinicaltrials.gov/.

**Supplementary Information:**

The online version contains supplementary material available at 10.1186/s12877-025-05979-6.

## Background

As the population ageing accelerates in many societies globally, there is an increasing need for promoting healthy ageing. Different modifiable lifestyle factors such as poor dietary habits, low physical activity, excessive alcohol use, smoking, and the presence of metabolic risk factors have been identified as the leading health risks for older people [1]. The Finnish Geriatric Intervention Study to Prevent Cognitive Impairment and Disability (FINGER) [2], originally aiming at preventing cognitive decline, was the first study to show that with an intensive multidomain lifestyle intervention, it is possible to promote healthy lifestyle and decrease the risk for cognitive and physical decline and multimorbidity among older people at increased risk for dementia [3–6]. While there is evidence indicating that lifestyle-based controlled trials have had favourable effects on people’s health behaviour during the intervention period [7–11], less is known about whether interventions also have long-term effects on lifestyle. There are studies reporting that some positive changes in lifestyle (e.g. dietary habits, physical activity, smoking, and alcohol consumption) achieved during the interventions were sustained during the extended post-intervention follow-up, but the length of follow-up periods have sometimes been short and results contradictory [10–15]. Studies reporting results from long-lasting follow-ups especially targeted at older people are scarce.

Ageing and temporal trends may cause changes in lifestyle over time. Changes may also occur as results of changes in the living environment such as the COVID-19 pandemic and its control measures. The COVID-19 pandemic has indisputably had severe impact on people of all ages, but especially on older people who were identified as a risk group for severe COVID-19 infections [16]. To control the spreading of the virus during the first wave of the pandemic in 2020, many countries adopted social distancing measures especially targeted to older people. Restricting older people’s possibilities for versatile activity became a concern during the pandemic.

To date, increasing number of studies have investigated on how the pandemic and its control measures have affected older people’s everyday lives and behaviours [17–24]. Many findings from the COVID-19 era have been based on cross-sectional surveys, self-evaluations of changes in lifestyle and behaviours, or longitudinal settings over a short period. It has remained unclear to what extent the reported situations truly were due to the pandemic, as there has been no pre-pandemic data to compare to. The extended follow-up of the FINGER intervention trial was ongoing during the COVID-19 pandemic and thus a survey during the first wave of pandemic provides a unique opportunity to put these changes in the context of long-term changes overall.

The aim of the present study was to investigate how multiple lifestyle factors and self-evaluated health have changed over a ten-year period in a cohort of older Finnish people at risk for developing dementia and participating in the FINGER trial. More specifically, objectives were to evaluate whether participants demonstrated changes in risk factors relevant for the prevention of cognitive decline and dementia, including social, cognitive, and physical activity, alcohol consumption, smoking, and dietary habits, and if such changes during the COVID-19 pandemic differed from the overall longitudinal trends.

## Methods

### Study design and participants

The design and recruitment process of the FINGER trial have been described in more detail previously [2, 3]. Briefly, the FINGER was a two-year multidomain lifestyle intervention trial (ClinicalTrials.gov NCT01041989, registered 04/01/2010) conducted in six areas in Finland starting in 2009. The study was population-based comprising of community-dwelling older participants aged 60–77 years at the beginning of the study and manifesting an elevated risk for developing dementia based on the CAIDE (Cardiovascular Risk Factors, Aging and Dementia) dementia risk score. In addition, to be included in the study, participants had to cognitively perform at an average level or slightly poorer in the CERAD (Consortium to Establish a Registry for Alzheimer’s Disease) test battery than expected for their age. Key exclusion criteria included previously diagnosed or suspected dementia and disorders affecting safe engagement in the intervention (e.g. malignant disease or major depression).

### Randomization and masking

The detailed trial design has been reported previously [2, 3]. Briefly, participants were randomized either into a multidomain lifestyle intervention group or a regular health advice group (control group) with a 1:1 allocation ratio. The study nurse did the computer-generated allocation in blocks of four at each study site after baseline. Double-blinding was pursued as completely as possible in lifestyle interventions.

### Procedures

The intervention contained four domains: dietary counselling, exercise training, cognitive training, and management of cardiovascular and metabolic risk factors [2]. Dietary counselling, based on Finnish Nutrition Recommendations, was conducted by nutritionists and included three individual and seven to nine group sessions. Exercise training was guided by physiotherapists and consisted of individualized and group training including progressive muscle strength training (1–3 times a week) and independent aerobic exercise (2–5 times a week). Cognitive training, focusing on executive processes, memory, and mental speed, was led by psychologists and consisted of ten group sessions and two periods of individual computer-based training (each period six months; 72 training sessions per period). Management of cardiovascular and metabolic risk factors included extra visits with the study nurse (at 3, 9, and 18 months) and the study physician (at 3, 6, and 12 months) for measurements, physical examinations, and recommendations for lifestyle management.

The control group received regular health advice meaning that they had visits with the study nurse (at screening, baseline, 6, 12, and 24 months) and the study physician (at screening and 24 months) for measurements and physical examination. At baseline, they also received oral and written information and advice on healthy diet and physical, cognitive, and social activities for supporting healthy aging. All these visits, information, and advice were provided also to the intervention group.

The intensive intervention period lasted for two years for each participant (during 2009–2014). Post-intervention follow-up examinations took place at 5 (during 2015-2016) and 7 years (during 2017-2018) after the baseline visit. During 2016-2018, text messages reminding of healthy lifestyles (e.g. tips on social, cognitive, and physical activities and healthy diet) were sent weekly to the intervention group. The 10-year follow-up was postponed due to COVID-19 pandemic, and thus a postal survey with questions related to the COVID-19 pandemic and lifestyle during the pandemic was developed and mailed to the participants in June 2020 [24].

### Outcomes

The primary outcome of the FINGER study was cognitive performance and results have been reported previously [3]. Present paper reports ancillary outcomes from the extended follow-up of the trial. Outcomes of this study were self-reported lifestyle measures representing physical, social, and cognitive activity, food consumption, alcohol use, smoking, and additionally self-evaluated health and memory. Most measures were available from all years (years 0, 1, 2, 5, 7, and the pandemic period), but food consumption questions were not included in the postal survey during the pandemic.

Physical activity was reported with a 7-point scale assessing frequency of at least moderate-intensity activity of at least 20 minutes at the time, ranging from 5 times a week or more to less than once a week. Those reporting inability to physical activity due to injury or illness were excluded from the analyses. Frequency of social and cognitive activities were collected using a 5-point scale (from daily to never). For the analyses, the scale was converted into times per week. Cognitive activity was calculated as the sum of reading, doing crosswords, writing, studying, practicing music, and making handicrafts; social activity was the sum of playing games, attending clubs and associations, babysitting, and taking part to volunteer work.

Frequencies for consumption of fruits and berries and vegetables and roots were reported with 6-point scales ranging from 6 portions or more per day to less than one portion per week. Examples for portions were given, for example a piece of fruit, a small portion of salad, or a handful of chopped vegetables. Fish consumption was assessed in weekly frequency of main dishes (e.g. fish, red meat, and poultry dishes) and participants were allowed to record their weekly frequency without pre-specified options.

Alcohol consumption was evaluated with two questions: frequency of any consumption (without specifying the amount) was collected by an 8-point scale (from daily to never) and binge drinking with a question concerning frequency of drinking at least 6 portions at once by a 7-point scale from daily to never. Examples for portions given were for example a small beer or glass of wine. For smoking, participants reported if they were smoking regularly, occasionally, or not at all. For analyses, smoking was categorized to smoking at least occasionally and not smoking. Current self-evaluated health and memory were reported with 5-point scales ranging from excellent to very poor.

To improve the interpretation, all the frequency related scales were inverted so that the bigger value referred to more frequent behaviour, and the health-related scales so that the bigger value referred to better health.

### Statistical analysis

We analysed numerical outcomes (fish intake and scores of social and cognitive activity) using linear mixed-effects regression model with restricted maximum likelihood estimation for all the endpoints. For ordinal outcomes (physical activity, vegetable and root consumption, fruit and berry consumption, alcohol use frequency, binge drinking, self-evaluated health and memory), we used ordinal logistic models. The ordinal outcomes were analysed using generalized estimating equations (GEE) with the R package “survey”. Missing data due to dropout was handled using inverse probability weights using the interaction of measurement point and treatment group, and sex, age, education, and marital status as predictors of participation.

We applied Type II ANOVA tests to assess the significance of main effects and/or interactions for the continuous outcomes and Wald tests for the ordinal outcomes. After these tests, to provide more detailed information about differences between specific study groups, multiple comparison analysis tests were used.

Regression analyses were adjusted for age at baseline, sex, self-reported education (in years), and marital status at baseline (dichotomized to living with someone, married or unmarried vs. living alone). We present the contrasts to compare both changes over time within groups with respect to the baseline, and differences between groups at each assessment. We present the standard errors and *p*-values of these contrasts. R 4.2 and RStudio 2022.02.1 were used for all the analyses. We used a level of significance of less than 5% in all the analyses.

## Results

### Characteristics and participation

**Fig. 1:**
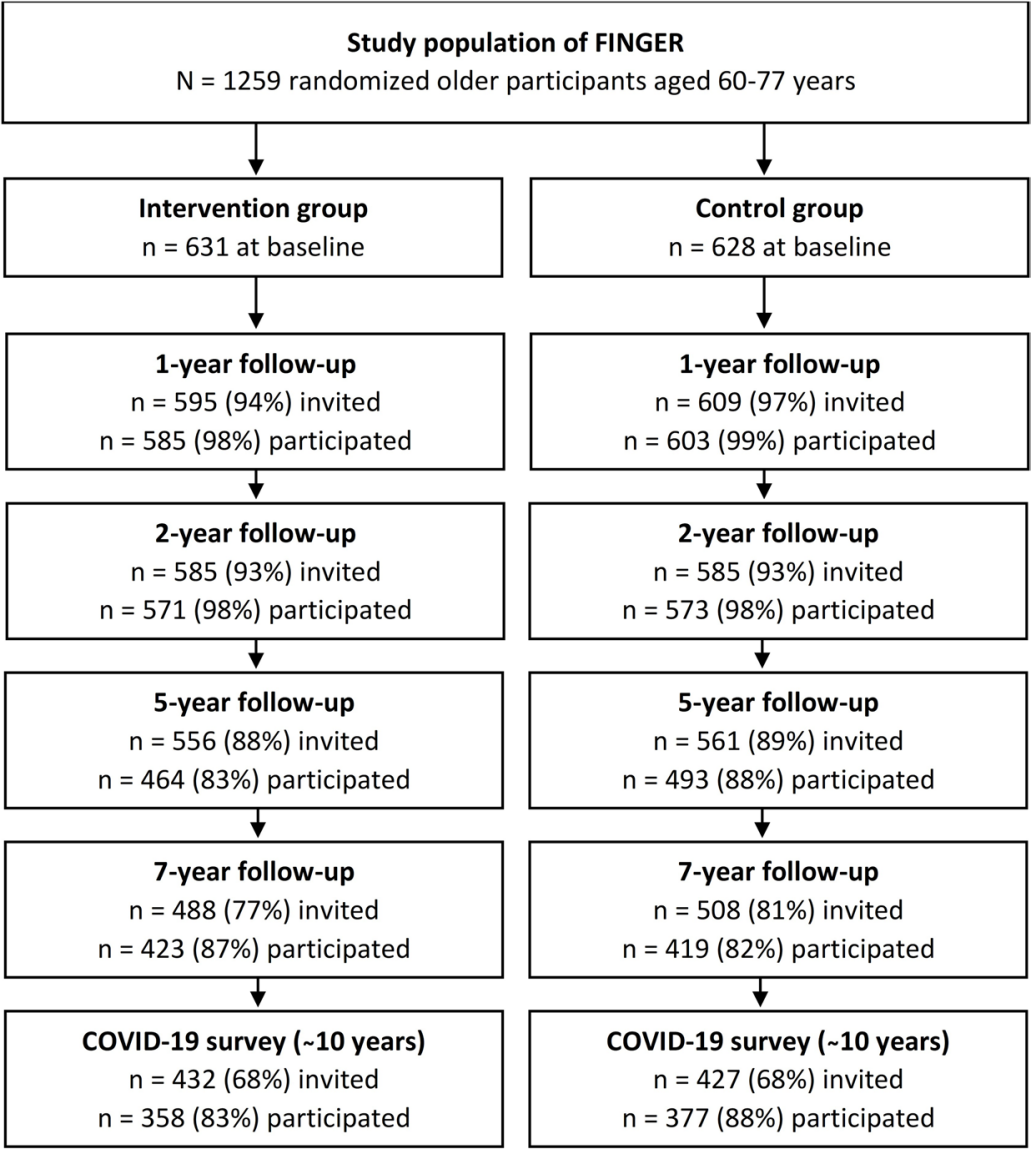
Flow chart of participation in the FINGER trial and its long-term follow-up For calculating percentages, the number of the people invited is divided by baseline value, and the number of participants by the number of the people invited

Participation in the study at different time points is presented in Fig. 1; 1259 subjects engaged in at baseline (631 intervention, 628 control), 1144 (91 %) after the intervention at 2 years, 842 (67 %) at the last clinical visit at 7 years, and 735 (58 %) at the postal survey during the pandemic. A total of 200 people were deceased during the follow-up (until September 2020). Altogether 859 participants (68 % of the original cohort) were eligible (alive and not withdrawn participation) for invitation to the COVID-19 survey, and 86 % of them responded. Baseline characteristics of those participating in different time points are presented in Table 1. Characteristics of those who dropped out at each visit are presented in Additional file 2, together with more information of baseline lifestyles among these two groups. As the follow-up proceeded, those who still participated were more often married or cohabiting, younger, more educated, and less often had diabetes at baseline compared to those who dropped out (Table 1). They were also less likely to have smoked at baseline and more likely to report good or very good memory and overall health (Additional file 2, *p*<0.05 for each comparison). Baseline characteristics of intervention and control groups did not differ from each other at any time points (Table 1, *p*>0.05 for each comparison).

**Table 1:** Selected baseline characteristics of the participants at different time points

	**Study group**	**Baseline (*n*=1259)**	**1 st year (*n*=1188)**	**2nd year (*n*=1144)**	**5 th year (*n*=957)**	**7 th year (*n*=842)**	**Pandemic**^**a**^** (*n*=735)**
**Baseline age (years), mean (SD**)	All	68.8 (4.7)	68.8 (4.7)	68.7 (4.7)	68.6 (4.7)**	68.5 (4.7)***	68.1 (4.7)***
	Intervention	69.0 (4.7)	69.0 (4.6)	68.9 (4.6)	68.7 (4.6)	68.6 (4.7)	68.4 (4.7)
	Control	68.7 (4.7)	68.7 (4.7)	68.5 (4.7)	68.6 (4.8)	68.3 (4.7)	67.8 (4.6)
**Men, n (%)**	All	672 (53.4%)	634 (53.4%)	614 (53.7%)	515 (53.8%)	447 (53.1%)	388 (52.8%)
	Intervention	345 (54.7%)	320 (54.7%)	317 (55.5%)	263 (56.7%)	234 (55.3%)	195 (54.5%)
	Control	327 (52.1%)	314 (52.1%)	297 (51.8%)	252 (51.1%)	213 (50.8%)	193 (51.2%)
**Education (years), mean (SD)**	All	10.0 (3.4)	10.0 (3.4)	10.0 (3.4)	10.1 (3.5)*	10.2 (3.5)**	10.2 (3.4)*
	Intervention	10.0 (3.5)	10.0 (3.4)	10.0 (3.4)	10.1 (3.5)	10.1 (3.6)	10.0 (3.3)
	Control	10.0 (3.4)	10.0 (3.4)	10.1 (3.4)	10.1 (3.4)	10.2 (3.4)	10.3 (3.5)
**Married orcohabiting at baseline, n (%)**	All	932 (74.4%)	883 (74.8%)	858 (75.5%)**	729 (76.6%)**	645 (77.1%)**	564 (77.2%)**
	Intervention	459 (73.3%)	430 (74.1%)	425 (75.1%)	357 (77.4%)	323 (76.9%)	274 (77.2%)
	Control	473 (75.6%)	453 (75.4%)	433 (75.8%)	372 (75.8%)	322 (77.2%)	290 (77.1%)
**Hypertension**^**b**^ **at baseline, n (%)**	All	647 (51.7%)	612 (51.9%)	591 (51.9%)	479 (50.4%)	415 (49.6%)*	363 (49.7%)
	Intervention	324 (51.6%)	302 (51.9%)	294 (51.7%)	230 (49.8%)	207 (49.2%)	174 (48.7%)
	Control	323 (51.8%)	310 (51.8%)	297 (52.2%)	249 (50.9%)	208 (50.0%)	189 (50.5%)
**Diabetes**^**b**^ **at baseline, n (%)**	All	168 (13.4%)	157 (13.3%)	149 (13.1%)	120 (12.6%)	100 (11.9%)*	83 (11.3%)*
	Intervention	87 (13.8%)	78 (13.4%)	74 (13.0%)	58 (12.5%)	51 (12.1%)	43 (12.0%)
	Control	81 (13.0%)	79 (13.2%)	75 (13.2%)	62 (12.7%)	49 (11.8%)	40 (10.7%)
**Depression**^**b**^ **at baseline, n (%)**	All	80 (6.4%)	78 (6.6%)	72 (6.3%)	57 (6.0%)	47 (5.6%)	41 (5.6%)
	Intervention	36 (5.7%)	35 (6.0%)	31 (5.4%)	24 (5.2%)	22 (5.2%)	19 (5.3%)
	Control	44 (7.1%)	43 (7.2%)	41 (7.2%)	33 (6.8%)	25 (6.0%)	22 (5.9%)

Changes reflect differences in participation in different time points. Statistically significant differences between participants and those who dropped out at different time points are presented in superscripts (*p<0.05, **p<0.01, ***p<0.001)

^a^Questionnaire in summer 2020, follow-up time on average 10 years

^b^Hypertension, diabetes, and depression were self-reported by participants at baseline

### General trends over time without the intervention

**Fig. 2:**
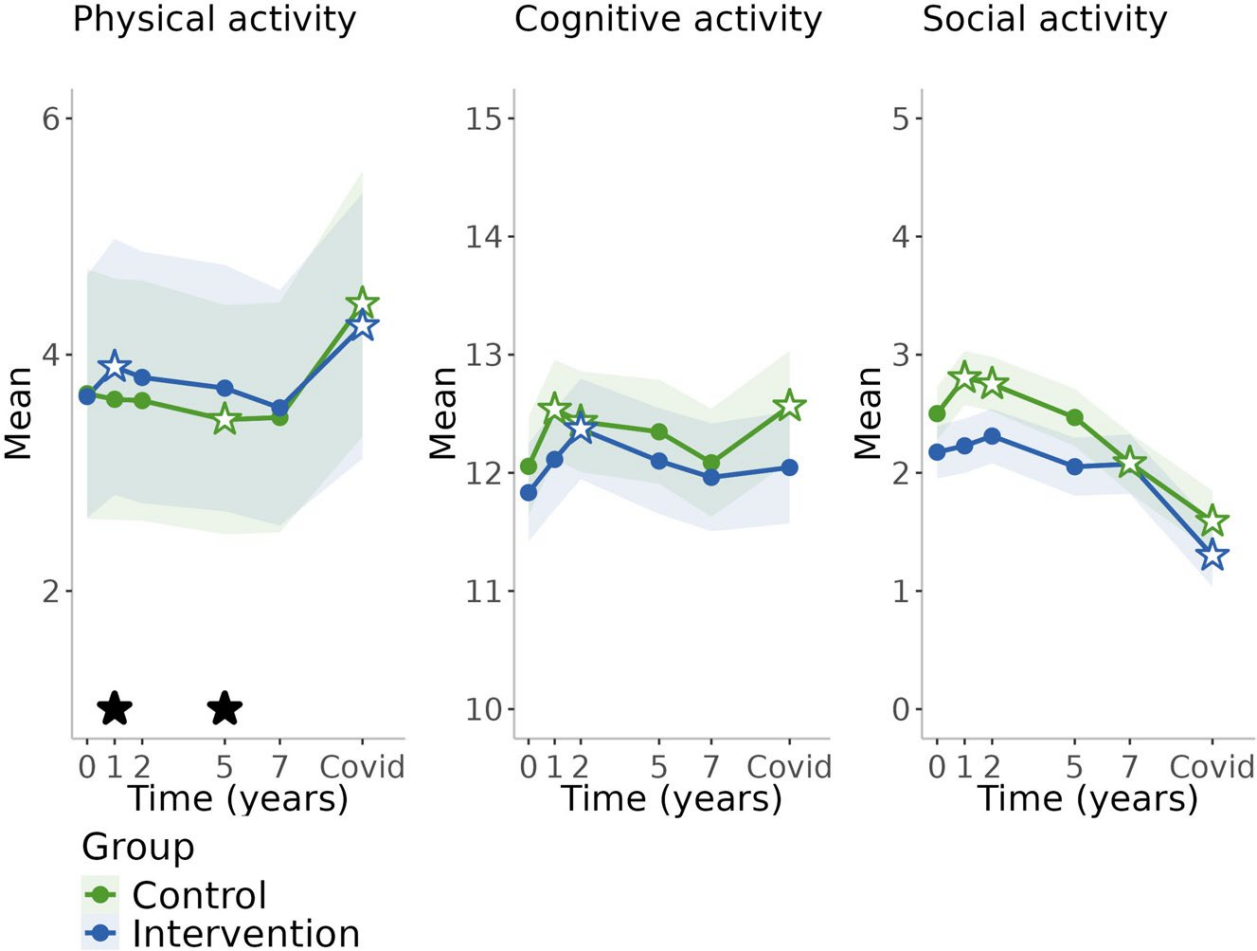
Changes in activities during the 10-year follow-up Black stars refer to statistically significant difference (*p*<0.05) between the groups, and white stars with blue/green lines refer to statistically significant difference from baseline within the group (with respective colour)

**Fig. 3:**
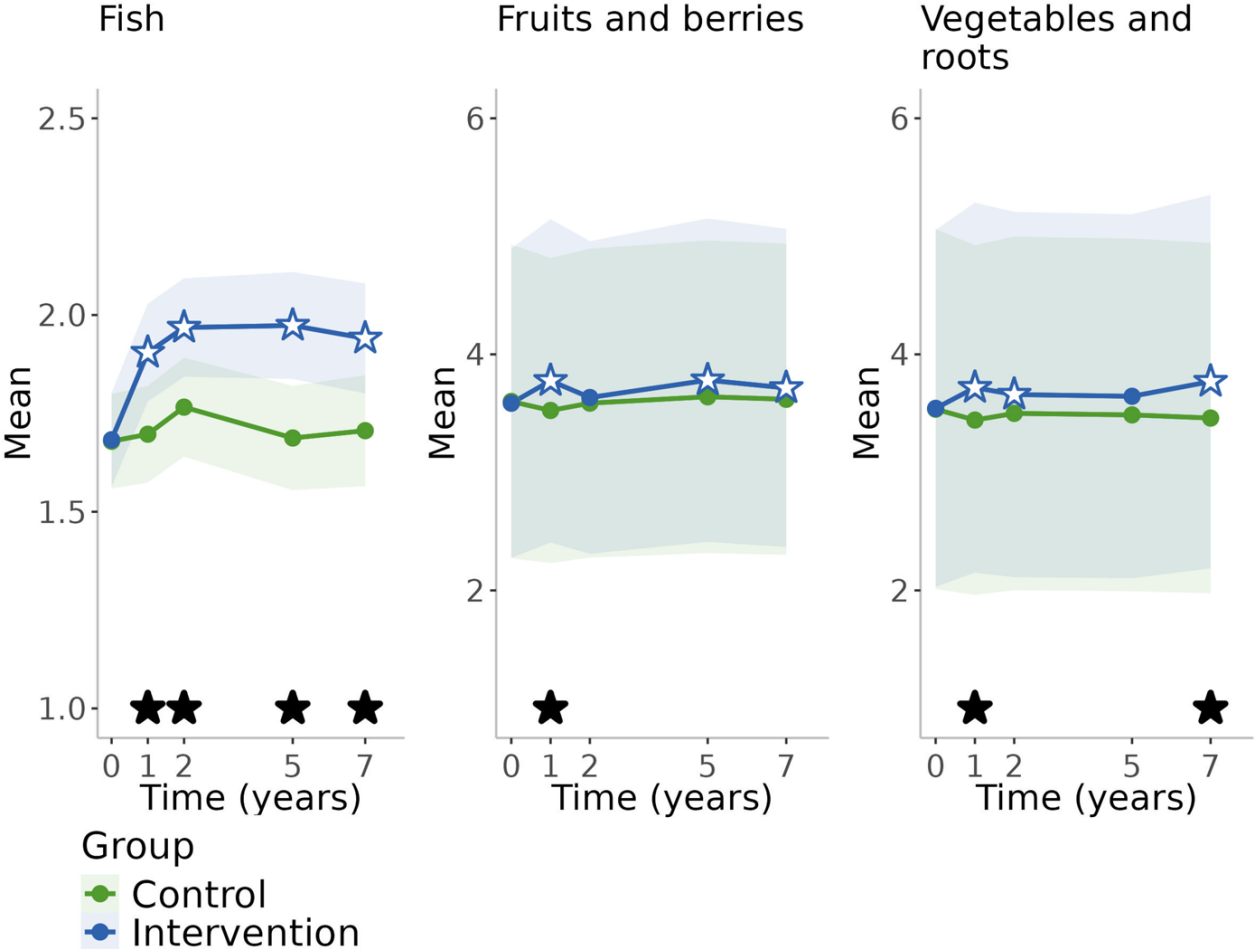
Changes in food consumption during the 7-year follow-up Black stars refer to statistically significant difference (*p*<0.05) between the groups, and white stars with blue/green lines refer to statistically significant difference from baseline within the group (with respective colour)

**Fig. 4:**
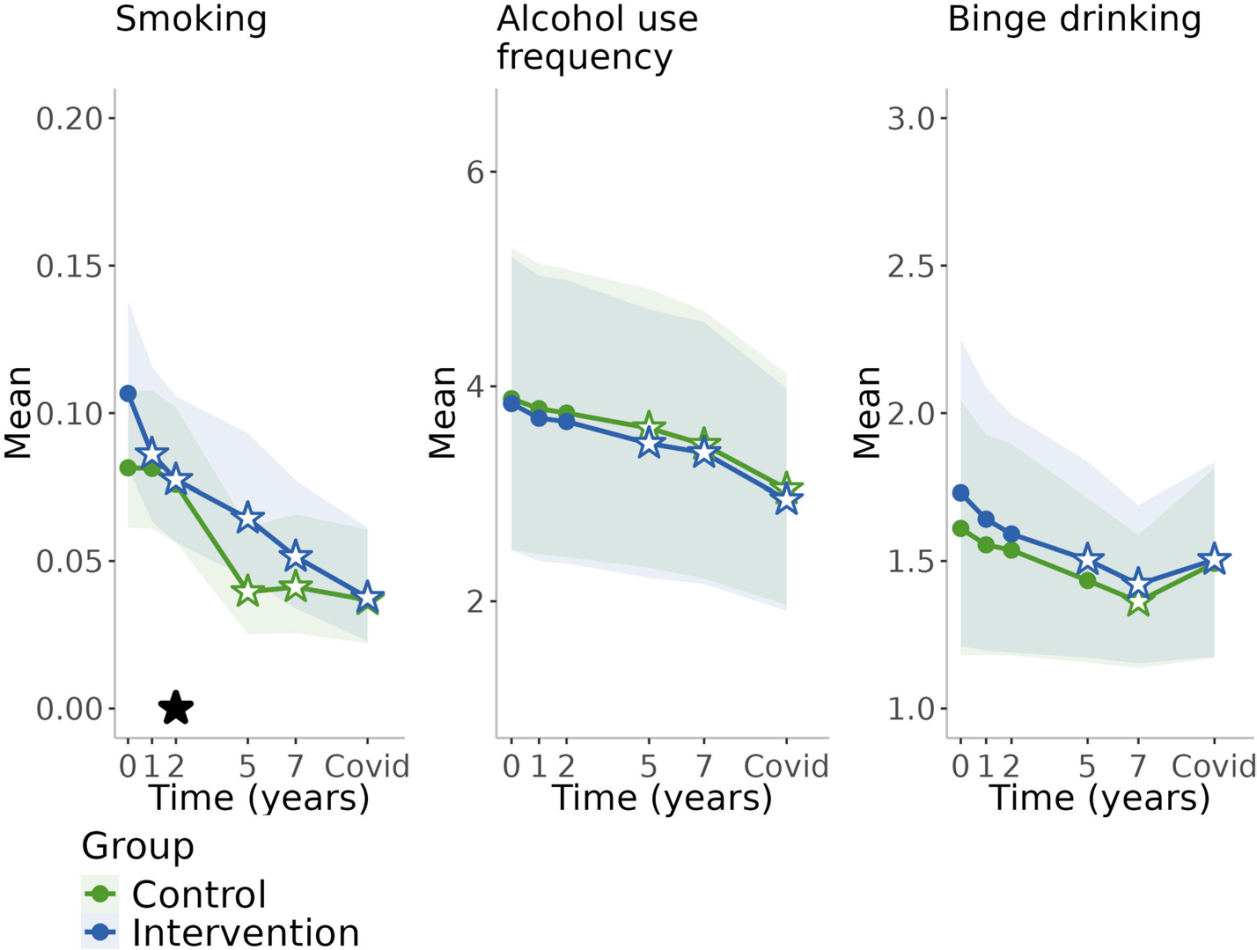
Changes in smoking and alcohol use during the 10-year follow-up Black star refers to statistically significant difference (*p*<0.05) between the groups, and white stars with blue/green lines refer to statistically significant difference from baseline within the group (with respective colour)

**Fig. 5:**
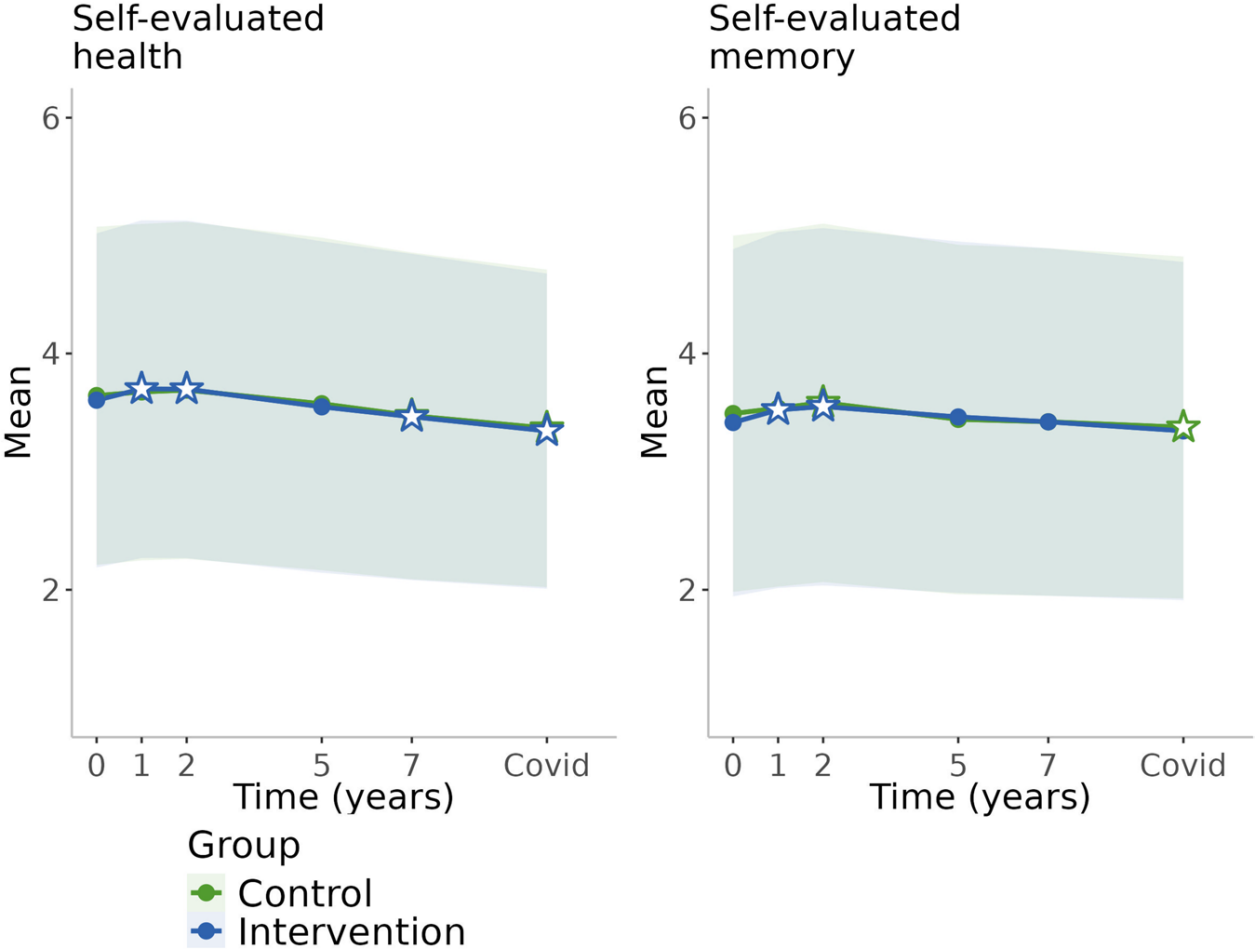
Changes in self-evaluated health during the 10-year follow-up White stars with blue/green lines refer to statistically significant difference (*p*<0.05) from baseline within the group (with respective colour)

Changes in lifestyle factors in both intervention and control groups during the follow-up period are presented in Table 2 and in Figs. 2, 3, 4 and 5. Crude values showing the level of lifestyle variables in the entire cohort (groups combined) are available in Additional file 1. In the control group, physical activity remained at the baseline level during the first two years (the active study period) after which it decreased until increased during the pandemic (Table 2, Fig. 2). Cognitive activity increased during the first year, decreased thereafter, until increased again during the pandemic. Social activity increased during the first year and decreased thereafter. Consumption of fish, vegetables and roots, and fruits and berries remained at the baseline level over time (Table 2, Fig. 3). Smoking remained at the baseline level during the active study period and decreased thereafter (Table 2, Fig. 4). There was decreasing trend in alcohol consumption frequency during the entire follow-up. Binge drinking decreased over time until the pandemic when it increased. Self-evaluated health remained at the baseline level and memory slightly improved during the active study period and declined thereafter (Table 2, Fig. 5).

**Table 2:** Temporal changes in lifestyle variables in the different groups and differences between the groups

	**Intervention**			**Control**			**Difference between groups (intervention-control)**		
	Contrast	SE	*p*-value	Contrast	SE	*p*-value	Contrast	SE	*p*-value
**Physical activity**^**a**^									
1y-Baseline	0.250	0.098	0.010	−0.047	0.095	0.620	0.297	0.141	0.034
2y-Baseline	0.161	0.095	0.090	−0.058	0.095	0.540	0.219	0.138	0.112
5y-Baseline	0.071	0.096	0.460	−0.220	0.104	0.035	0.290	0.144	0.044
7y-Baseline	−0.096	0.097	0.323	−0.201	0.104	0.053	0.105	0.137	0.445
Pandemic-Baseline	0.597	0.106	<0.001	0.762	0.096	<0.001	−0.165	0.130	0.205
**Cognitive activity**^**b**^									
1y-Baseline	0.281	0.180	0.118	0.482	0.177	0.007	−0.201	0.253	0.427
2y-Baseline	0.539	0.182	0.003	0.379	0.181	0.037	0.160	0.256	0.532
5y-Baseline	0.268	0.196	0.173	0.293	0.192	0.127	−0.025	0.274	0.928
7y-Baseline	0.129	0.202	0.523	0.030	0.203	0.881	0.099	0.286	0.729
Pandemic-Baseline	0.213	0.214	0.320	0.509	0.212	0.016	−0.296	0.299	0.322
**Social activity**^**b**^									
1y-Baseline	0.053	0.110	0.637	0.302	0.109	0.005	−0.249	0.155	0.108
2y-Baseline	0.136	0.111	0.223	0.250	0.111	0.025	−0.114	0.157	0.468
5y-Baseline	−0.123	0.120	0.304	−0.032	0.117	0.782	−0.091	0.168	0.588
7y-Baseline	−0.101	0.124	0.414	−0.417	0.124	0.001	0.316	0.175	0.071
Pandemic-Baseline	−0.875	0.131	<0.001	−0.913	0.129	<0.001	0.037	0.183	0.839
**Fish**^**b**^									
1y-Baseline	0.222	0.068	0.001	0.018	0.067	0.788	0.204	0.095	0.033
2y-Baseline	0.285	0.069	<0.001	0.087	0.069	0.205	0.198	0.097	0.041
5y-Baseline	0.290	0.074	<0.001	0.008	0.072	0.908	0.282	0.103	0.006
7y-Baseline	0.258	0.076	<0.001	0.027	0.077	0.724	0.231	0.108	0.033
**Fruits and berries**^**a**^									
1y-Baseline	0.190	0.064	0.003	−0.076	0.061	0.208	0.267	0.094	0.005
2y-Baseline	0.048	0.057	0.398	−0.014	0.058	0.805	0.063	0.083	0.448
5y-Baseline	0.195	0.064	0.002	0.040	0.057	0.486	0.156	0.086	0.071
7y-Baseline	0.129	0.060	0.030	0.018	0.057	0.747	0.111	0.084	0.186
**Vegetables and roots**^**a**^									
1y-Baseline	0.175	0.062	0.004	−0.092	0.059	0.120	0.268	0.093	0.004
2y-Baseline	0.117	0.058	0.043	−0.036	0.057	0.534	0.153	0.084	0.070
5y-Baseline	0.102	0.057	0.070	−0.048	0.058	0.400	0.151	0.084	0.071
7y-Baseline	0.227	0.066	<0.001	−0.075	0.059	0.204	0.302	0.096	0.002
**Smoking**^**a**^									
1y-Baseline	−0.237	0.088	0.007	−0.002	0.095	0.980	−0.234	0.129	0.070
2y-Baseline	−0.353	0.104	0.001	−0.075	0.094	0.426	−0.278	0.139	0.046
5y-Baseline	−0.550	0.151	<0.001	−0.767	0.191	<0.001	0.217	0.243	0.373
7y-Baseline	−0.789	0.182	<0.001	−0.725	0.199	<0.001	−0.064	0.267	0.812
Pandemic-Baseline	−1.118	0.238	<0.001	−0.843	0.225	<0.001	−0.275	0.325	0.398
**Alcohol use**^**a**^									
1y-Baseline	−0.137	0.103	0.185	−0.093	0.105	0.378	−0.044	0.144	0.759
2y-Baseline	−0.168	0.104	0.104	−0.134	0.107	0.211	−0.035	0.143	0.808
5y-Baseline	−0.373	0.117	0.001	−0.274	0.116	0.018	−0.099	0.143	0.488
7y-Baseline	−0.460	0.126	<0.001	−0.427	0.131	0.001	−0.032	0.143	0.821
Pandemic-Baseline	−0.895	0.196	<0.001	−0.841	0.196	<0.001	−0.055	0.142	0.700
**Binge drinking**^**a**^									
1y-Baseline	−0.089	0.065	0.172	−0.056	0.058	0.334	−0.033	0.072	0.646
2y-Baseline	−0.139	0.079	0.079	−0.073	0.061	0.230	−0.065	0.074	0.375
5y-Baseline	−0.227	0.108	0.036	−0.177	0.091	0.053	−0.050	0.070	0.476
7y-Baseline	−0.309	0.137	0.024	−0.248	0.115	0.031	−0.061	0.069	0.375
Pandemic-Baseline	−0.226	0.109	0.038	−0.118	0.073	0.108	−0.108	0.079	0.173
**Self-evaluated health**^**a**^									
1y-Baseline	0.095	0.040	0.017	0.031	0.039	0.428	0.065	0.056	0.253
2y-Baseline	0.094	0.040	0.019	0.046	0.039	0.233	0.048	0.056	0.396
5y-Baseline	−0.056	0.040	0.160	−0.069	0.041	0.095	0.013	0.056	0.819
7y-Baseline	−0.140	0.044	0.001	−0.171	0.047	<0.001	0.030	0.057	0.597
Pandemic-Baseline	−0.261	0.058	<0.001	−0.276	0.061	<0.001	0.015	0.058	0.791
**Self-evaluated memory**^**a**^									
1y-Baseline	0.107	0.042	0.011	0.046	0.037	0.220	0.062	0.056	0.273
2y-Baseline	0.136	0.044	0.002	0.093	0.038	0.013	0.042	0.056	0.444
5y-Baseline	0.047	0.039	0.228	−0.049	0.040	0.225	0.096	0.059	0.101
7y-Baseline	0.006	0.038	0.884	−0.070	0.043	0.101	0.075	0.057	0.189
Pandemic-Baseline	−0.071	0.043	0.101	−0.116	0.049	0.019	0.045	0.056	0.418

Changes in lifestyle variables in the intervention and control groups, and differences between the groups during the active study period (up to 2 years) and post-intervention follow-up (up to the COVID-19 pandemic). Contrasts with their standard errors and *p*-values are presented; positive values refer to increase and negative values to decrease

^a^Generalized estimating equations (GEE) used in analysis

^b^Linear mixed-effects regression model used in analysis

### Differences between the intervention and control group over the 10-year study period

Participants in the intervention group increased physical activity during the first year of the intervention and were physically more active than the control group at the first and the fifth year of the follow-up (Table 2, Fig. 2). After that, there was no difference between the groups. Consumption of fish was more abundant in the intervention group throughout the follow-up period (Table 2, Fig. 3) and consumption of vegetables and roots was above baseline level in the intervention group over time resulting statistically significant differences between the groups at the first and the seventh year of follow-up. Consumption of fruits and berries were above the baseline level at every study point except for the second year of the follow-up. There was a statistically significant difference between the groups only at the first year of the follow-up. Smoking decreased in both groups over time, but the decrease was steeper in the intervention group during the active study period resulting in a significant difference between the groups at the second year of the follow-up but no differences thereafter (Table 2, Fig. 4). There were no group differences in changes in social and cognitive activities, alcohol consumption, and self-evaluated health and memory (Table 2, Figs. 2, 4 and 5).

### Changes during the pandemic

Physical activity increased from the 7-year visit to the pandemic study point (*p*<0.001 in the control group; *p*<0.001 in the intervention group), which was of opposite to the decline in physical activity detected during the follow-up prior the pandemic. This increase was higher in the control group (*p*=0.041 for group difference). Similar trend was detected for cognitive activity, which was higher during the pandemic compared with the 7-year time point especially in the control group (within-group *p*=0.034), although there had been some decline in these activities prior the pandemic. However, changes between the 7-year visit and the pandemic time point were not different between the groups for cognitive activity (Comparison with baseline shown in Table 2 and Fig. 2; comparison with previous visits in Additional file 3).

In line with earlier trend, alcohol use frequency decreased, but on the contrary, binge drinking increased or remained from the 7-year visit to the pandemic (*p*=0.043 in the control group; *p*=0.143 in the intervention group) (Table 2, Fig. 4, Additional file 3). A decrease was found in social activity and smoking as well as in self-evaluated health and memory during the pandemic compared with baseline; these trends were in line with trends during the previous years but changes in smoking and memory were not statistically significant between the 7-year visit and the pandemic (Table 2, Figs. 2, 4, 5, Additional file 3). Men and those living alone were more likely to experience decline in cognitive activity during the pandemic (characteristic*time interaction *p*<0.05), but there were no other differences between the subgroups.

## Discussion

In this study, multidomain lifestyle intervention resulted in favourable changes in participants’ dietary habits and physical activity, and part of the improvements remained also during the long-term post-intervention follow-up. Although no significant differences between the intervention and control groups were evident in other modifiable factors, we showed that, in the whole cohort, social and cognitive activities increased, and self-evaluated health and memory improved during the active study period but decreased thereafter. Alcohol use tended to decrease over time. Smoking decreased more in the intervention group during the intervention period, but this difference was not maintained in longer term. During the COVID-19 pandemic, frequency of participants’ physical and cognitive activities increased and so did binge drinking.

In line with previous studies [7–9], physical activity increased, and diet improved during the intervention in the intervention group. The effect of the intervention on physical activity was no longer observed during the long-term post-intervention follow-up, but improvements in diet achieved during the intervention were mainly sustained. These results are fairly similar to those from the Finnish Diabetes Prevention Study targeting lifestyle intervention to overweight, middle-aged persons with impaired glucose tolerance; favourable effects of intervention on dietary habits and weight were sustained, but not the effects on physical activity in the long-term post-interventional follow-up period [12]. Not many earlier studies have reported post-intervention sustenance results, but in our study the main reason for this difference is most likely the set-up of the original intervention. During the original intervention period free access and instruction to gym was provided, but afterwards participants were required to conduct and also pay their training independently. Original intervention included support to plan how to continue training individually, but better integration of the training option provided by community and other stakeholders could have improved the long-term results in physical activity. In dietary intervention, on the other hand, the main content during the original intervention was learning to adopt healthy diet independently at home, which is likely to be better maintained after the supported period is over. Also, as our maintenance intervention consisted of text messages to provide small tips and reminders, and may thus have been more suited to support smaller changes adopted at home.

Frequency of cognitive and social activities increased, and self-evaluated health and memory improved during the active study period, but the changes were largely similar in both groups. These changes may be partly associated with the participation in the study, i.e. Hawthorne effect. While people in the intervention group had many individual and group intervention sessions, the control group similarly visited the study nurse and physician several times during the first two years. During the study visits, the study nurse gave all participants oral and written information and advice on healthy lifestyle (diet, physical, cognitive, and social activities) for supporting healthy aging. It is a typical finding in lifestyle interventions that the active study period has effects on health behaviour on both intervention and control groups [11, 12, 14].

A decrease in social activity has been shown to be associated with cognitive decline [25]. In our study, social activity decreased in post-intervention follow-up, and the pandemic did not notably influence such trend in older people at risk of developing dementia. A Finnish study [26] investigating the effect of the pandemic on activity in older people reported that many kinds of activities (including social, cognitive, and physical) decreased during the pandemic. In a study from Australia, participants reported decreasing trend in social activities but an increase in cognitive activity during the COVID-19 pandemic [22]. In that study, the increase in cognitive activity was, however, at least partly associated with engagement in a public health program targeting dementia risk reduction rather than with the pandemic. In our study, cognitive activity increased during the pandemic as opposed to previous trend. It is possible that decrease in other activities outside home during the restrictions were partly compensated by cognitive activities, which can take place at home, such as reading, writing, or crosswords. This could indicate that people were able to adjust their activities in the restrictions and such flexibility may be important for coping with the situation.

In line with previous studies [19, 21–23], smoking was rare among older adults and reduced during the pandemic. Regarding alcohol use, consumption frequency decreased but frequency of binge drinking increased which has been found also in other high-income countries [27] and could be related to the polarization of drinking habits that has become more obvious during the pandemic [28]. Decline in social activities and limited access to places outside home can also possibly explain overall lower frequency of drinking.

The biggest contradicting finding, as opposed to other studies, was the change in physical activity during the COVID-19 pandemic period. In our study, frequency of physical activity increased significantly whereas in most of previous studies, physical activity has decreased [17–21, 29]. In many of those studies, participants have self-evaluated the change in physical activity (e.g. decreased, increased, remained the same) which may have led to the differences in our results where change in physical activity was measured as a change in physical activity level between different study points. Also in our COVID-19 survey participants evaluated how they felt their physical activity was changed (more, less, the same than before the pandemic), and we have previously reported that participants considered that their physical activity decreased [24], whereas we see an increase when analysing level reported before the pandemic and during the pandemic in this study. One reason for the discrepancy between subjective feeling of change and longitudinally measured change could be availability of other activities; as most gatherings and outside home activities were closed down during the pandemic (reflected also in social activities in this study), people had more free time at home. Thus, their experience of time spent in physical activities could have been lower simply due to more overall free time. In addition, the type of activity may have changed when gyms and most instructed exercise sessions were closed, but there was still possibility for going outdoors, and thus increased frequency may come with change in the type of sports and/or decreased intensity. Present study only measured moderate-intensity activity of at least 20 minutes at the time which is not likely to represent the total physical activity of the study participants. However, the used assessment is likely to capture various types of exercise conducted outdoors (e.g. walking and jogging) which were more accessible also during the pandemic and related restrictions.

Based on these results, the effect of the pandemic period on lifestyle was overall more positive than expected as we did not find any statistically significant negative changes between the 7-year time point and the pandemic; and we did observe increased frequency of physical activity. Older people are used to accommodate their activity because of declining health and function along with ageing [30] which may partly explain the results. Also, pandemic related changes in everyday life in older adults in our study group, who were mostly retired and still living independently at home, could have been smaller than for younger people who were used to going to school or work and meeting more people outside home; or for those older adults who were living in institutions outside home. It’s worth noting that this questionnaire was completed after the first wave of the pandemic with a relatively strict restrictions ongoing but still after the lockdown related measures were already lifted. People may have felt more positive after the most difficult period was over. Also, these changes represent short-term effects of the pandemic during its first 6 months and effects in longer term may be different. It would also be important to identify groups that were maybe more vulnerable to pandemic. In our earlier analysis with self-evaluated changes those living alone reported more negative changes, e.g. in physical activity and overall health, but these findings were not evident here with longitudinal data.

### Strengths and limitations

In the present study, we reported results from long-term follow-up (ten years) of the multidomain intervention trial. This enabled us to assess the effect of the intervention on lifestyle during the active trial and also in long-term. We had longitudinal data on different outcomes from many years before the COVID-19 pandemic, and thus, we were able to compare the pandemic-associated changes to the preceding trends, not only to the data based on participants’ perceived changes that has been done in the most studies reporting pandemic-associated health changes and that is more prone to recall bias.

Methodological limitations include reliability of the self-report in outcomes, without objective measures. However, measures were mainly the same at every follow-up point which make them comparable to each other. The purpose of this study was to describe general trends in older people’s lifestyle with and without intervention and before and during the pandemic. Therefore, we reported changes in lifestyle using crude measures of different lifestyle factors which were also available from postal survey during the pandemic. More detailed analyses on changes in individual lifestyle factors with more specific measures taken during study visits and more individualised approach are forthcoming after the post-pandemic follow-up visits are completed. Future studies should involve more objective indicators, such as wearables for physical activity or biomarkers for dietary intake.

Another main limitation is the attrition. The study population participating at the 10-year follow-up was still relatively healthy and mostly independently living, because people engaging in lifestyle intervention and still participating after ten years are likely to be selected and may thus not represent all older adults in Finland, although the cohort is recruited from population-based sample. Participation rate among eligible participants was high throughout the study, but due to deaths, illnesses, and withdrawals only 60% were still included at the end of this follow-up. The original selection of at-risk group attenuates the typical selection of healthy and health-conscious people in studies, but cohort attrition is likely to influence the results in the latest visits. There were no differences between the original intervention and control groups in terms of attrition, making the group comparisons less likely biased, but likely that true changes over time would be more negative than in this selected population.

A pandemic-associated limitation is that the pandemic follow-up point was during the very first months after the pandemic started, right after the first wave although after most restrictions were already lifted. Based on these data, long-term consequences of the pandemic on lifestyle factors are not known. On one hand, it is also possible that lifestyle was more affected during this period as compared to later waves after vaccinations were introduced and restrictions were less strict. On the other hand, it’s also possible that people felt more optimistic after the most restricted period was over. Also, the questionnaire was sent during summer, which made it easier to move outdoors and meet people, when there were still restrictions on gatherings indoors. Originally participants came to the study visits different times of year.

## Conclusions

In summary, the multidomain lifestyle intervention aiming at preventing cognitive impairment and disability had some favourable longitudinal effects on participants’ lifestyle, especially on dietary intake. The COVID-19 pandemic had a few effects on lifestyle of older people at risk for developing dementia, especially increase in physical activity. In the future, it would be important to study how changes that were positive in short term but diluted in longitudinal follow-up would be maintained also in long term. In addition, it would be interesting to investigate if pandemic-related changes will sustain or vanish.

## Supplementary Information

Additional file 1. Crude values of lifestyle variables in the entire cohort.

Additional file 2. Characteristics of the participants and non-participants

Additional file 3. Pandemic related changes

## Data Availability

No datasets were generated or analysed during the current study.

## Abbreviations

ANOVA: Analysis of variance
CAIDE: Cardiovascular Risk Factors, Aging and Dementia risk score
CERAD: Consortium to Establish a Registry for Alzheimer’s Disease
COVID-19: Coronavirus disease
FINGER: The Finnish Geriatric Intervention Study to Prevent Cognitive Impairment and Disability
GEE: Generalized estimating equations
SE: Standard error
SD: Standard deviation
