# Supplementary material for: Changes in older persons’ lifestyle and perceived health over time and during the COVID-19 pandemic: findings from the extended follow-up of the FINGER randomized controlled trial from 2009 to 2020
Source: BMC Geriatr. 2025 May 3;25:308. doi: 10.1186/s12877-025-05979-6 (PMC12048948; doi:10.1186/s12877-025-05979-6)
Supplement: Supplementary file 1 — Additional file 1. Crude values of lifestyle variables in the entire cohort. [file 12877_2025_5979_MOESM1_ESM.pdf]

Supplementary table 1. Crude values of lifestyle variables in the entire cohort (intervention and control groups combined) throughout the follow-up period. N-values include those who were included into the analysis (those who had responded to the questionnaire including the analysed lifestyle variables).

	**Baseline** (N=1259[1])	**1st year** (N=1166[2])	**2nd year** (N=1118[3])	**5th year** (N=934[4])	**7th year** (N=813[5])	**Pandemic** (N=735[6])
**Cognitive activity** (times per week, mean, SD)	11.8 (5.0)	12.3 (5.5)	12.3 (5.2)	12.3 (5.3)	12.0 (5.4)	12.5 (6.0)
**Social activity** (times per week, mean, SD)	2.3 (2.8)	2.5 (2.9)	2.6 (3.0)	2.3 (2.7)	2.2 (2.7)	1.5 (2.4)
**Physical activity**, n (%)[7]						
Once a week or less	315 (26.3%)	267 (24.0%)	251 (23.6%)	241 (28.6%)	224 (31.3%)	104 (15.6%)
2 times per week	200 (16.7%)	174 (15.6%)	193 (18.1%)	149 (17.7%)	121 (16.9%)	88 (13.2%)
3 times per week	287 (24.0%)	278 (25.0%)	259 (24.3%)	184 (21.8%)	147 (20.6%)	115 (17.2%)
4 times per week or more	395 (33.0%)	393 (35.3%)	362 (34.0%)	270 (32.0%)	223 (31.2%)	361 (54.0%)
**Smokers**, n (%)	114 (9.4%)	97 (8.4%)	87 (7.8%)	50 (5.5%)	41 (5.1%)	31 (4.2%)
**Alcohol use**, n (%)[7]						
Less than monthly	438 (35.0%)	439 (37.7%)	421 (37.8%)	364 (40.1%)	364 (45.0%)	377 (51.9%)
Once or twice per month	257 (20.5%)	228 (19.6%)	216 (19.4%)	202 (22.2%)	157 (19.4%)	127 (17.5%)
Once a week or more	462 (36.9%)	422 (36.3%)	408 (36.7%)	292 (32.2%)	245 (30.3%)	203 (27.9%)
Daily	94 (7.5%)	75 (6.4%)	68 (6.1%)	50 (5.5%)	42 (5.2%)	19 (2.6%)
**Binge drinking**, n (%)[7]						
Never	701 (59.5%)	683 (62.7%)	669 (63.6%)	590 (68.4%)	562 (72.0%)	337 (63.1%)
Less than once per month	322 (27.3%)	291 (26.7%)	279 (26.5%)	196 (22.7%)	167 (21.4%)	141 (26.4%)
At least once per month	93 (7.9%)	69 (6.3%)	68 (6.5%)	44 (5.1%)	30 (3.8%)	37 (6.9%)
At least once per week	62 (5.3%)	47 (4.3%)	36 (3.4%)	33 (3.8%)	22 (2.8%)	19 (3.6%)
**Fish** (portions per week, mean, SD)	1.7 (1.1)	1.8 (1.5)	1.9 (2.1)	1.9 (1.2)	1.9 (1.2)	N/A
**Fruits and berries**, n (%)[7]						
Less than 3 portions per week	220 (17.5%)	178 (15.3%)	202 (18.1%)	130 (14.2%)	130 (16.0%)	N/A
4-6 portions per week	225 (17.9%)	210 (18.1%)	189 (17.0%)	160 (17.4%)	126 (15.6%)	N/A
1-2 portions per day	616 (49.0%)	574 (49.4%)	538 (48.3%)	451 (49.1%)	415 (51.2%)	N/A
3 or more portions per day	196 (15.6%)	199 (17.1%)	186 (16.7%)	177 (19.3%)	139 (17.2%)	N/A
**Vegetables and roots**, n (%)[7]						
Less than 3 portions per week	227 (18.1%)	175 (16.4%)	180 (17.2%)	157 (17.1%)	144 (17.8%)	N/A
4-6 portions per week	254 (20.2%)	224 (21.0%)	207 (19.8%)	180 (19.6%)	139 (17.2%)	N/A
1-2 portions per day	604 (48.1%)	497 (46.6%)	493 (47.2%)	454 (49.5%)	392 (48.5%)	N/A
3 or more portions per day	171 (13.6%)	170 (15.9%)	165 (15.8%)	126 (13.7%)	134 (16.6%)	N/A

**Self-evaluated health**, n (%)[7]						
Poor or very poor	43 (3.4%)	49 (4.2%)	46 (4.1%)	52 (5.7%)	73 (9.1%)	103 (14.3%)
Intermediate	459 (36.7%)	360 (30.9%)	330 (29.7%)	342 (37.5%)	313 (38.9%)	280 (38.8%)
Good or very good	750 (59.9%)	756 (64.9%)	735 (66.2%)	517 (56.8%)	418 (52.0%)	339 (47.0%)
**Self-evaluated memory**, n (%)[7]						
Poor or very poor	70 (5.6%)	49 (4.2%)	48 (4.3%)	69 (7.7%)	66 (8.2%)	83 (11.6%)
Intermediate	591 (47.0%)	501 (43.2%)	436 (39.4%)	391 (43.4%)	362 (45.1%)	317 (44.3%)
Good or very good	596 (47.4%)	611 (52.6%)	624 (56.3%)	441 (48.9%)	374 (46.6%)	315 (44.1%)

[1]Number of participants providing data for each lifestyle factor vary between 1178-1257.

[2]Number of participants providing data for each lifestyle factor vary between 1066-1166.

[3]Number of participants providing data for each lifestyle factor vary between 1045-1118.

[4]Number of participants providing data for each lifestyle factor vary between 844-918.

[5]Number of participants providing data for each lifestyle factor vary between 715-811.

[6]Number of participants providing data for each lifestyle factor vary between 534-731.

[7]Categories of the original question have been combined to facilite reading of the table (analyses conducted with the original categories).

N/A: not available.
