# Supplementary material for: Changes in older persons’ lifestyle and perceived health over time and during the COVID-19 pandemic: findings from the extended follow-up of the FINGER randomized controlled trial from 2009 to 2020
Source: BMC Geriatr. 2025 May 3;25:308. doi: 10.1186/s12877-025-05979-6 (PMC12048948; doi:10.1186/s12877-025-05979-6)
Supplement: Supplementary file 2 — Additional file 2. Characteristics of the participants and non-participants [file 12877_2025_5979_MOESM2_ESM.pdf]

Supplementary table 2. Baseline characteristics and dichotomised baseline lifestyles presented based on participation at different time point. Differences between participants (Ps) and non-participants (Non-ps) at each visit are presented and compared.

	Baseline	1st year		2nd year		5th year		7th year		Pandemic[1]	
	Ps **(n=1259)**	Ps (n=1188)	Non-ps (n=71)	Ps (n=1144)	Non-ps (n=115)	Ps (n=957)	Non-ps (n=302)	Ps (n=842)	Non-ps (n=417)	Ps (n=735)	Non-ps (n=524)
**Baseline age** (years), mean (SD)	68.8 (4.7)	68.8 (4.7)	69.0 (4.9)	68.7 (4.7)	69.8 (4.7)	68.6 (4.7)	69.5 (4.7)**	68.5 (4.7)	69.5 (4.6)***	68.1 (4.7)	69.8 (4.6)***
**Men,** n (%)	672 (53.4%)	634 (53.4%)	38 (53.5%)	614 (53.7%)	58 (50.4%)	515 (53.8%)	157 (52%)	447 (53.1%)	225 (54%)	388 (52.8%)	284 (54.2%)
**Education** (years), mean (SD)	10.0 (3.4)	10.0 (3.4)	9.6 (4.1)	10.0 (3.4)	9.7 (3.7)	10.1 (3.5)	9.5 (3.4)*	10.2 (3.5)	9.6 (3.3)**	10.2 (3.4)	9.7 (3.5)*
**Married or cohabiting** at baseline, n (%)	932 (74.4%)	883 (74.8%)	49 (69.0%)	858 (75.5%)	74 (64.3%)	729 (76.6%)	203 (67.7%)**	645 (77.1%)	287 (69.2%)**	564 (77.2%)	368 (70.6%)**
**Hypertension**[2] at baseline, n (%)	647 (51.7%)	612 (51.9%)	35 (49.3%)	591 (51.9%)	56 (49.6%)	479 (50.4%)	168 (56.0%)	415 (49.6%)	232 (56.0%)	363 (49.7%)	284 (54.6%)
**Diabetes**[2] at baseline, n (%)	168 (13.4%)	157 (13.3%)	11 (15.5%)	149 (13.1%)	19 (16.8%)	120 (12.6%)	48 (16.0%)	100 (11.9%)	68 (16.4%)*	83 11.3%)	85 (16.3%)*
**Depression**[2] at baseline, n (%)	80 (6.4%)	78 (6.6%)	2 (2.9%)	72 (6.3%)	8 (7.2%)	57 (6.0%)	23 (7.7%)	47 (5.6%)	33 (8.0%)	41 (5.6%)	39 (7.5%)
**Cognitive activity** (times per week, mean(SD)	11.8 (5.0)	11.8 (5.0)	12.1 (5.4)	11.8 (4.9)	12.3 (5.3)	11.8 (4.9)	11.9 (5.2)	11.9 (4.8)	11.8 (5.3)	12.0 (4.8)	11.7 (5.2)
**Social activity** (times per week), mean (SD)	2.3 (2.8)	2.4 (2.8)	1.8 (2.4)	2.4 (2.8)	2.1 (2.8)	2.4 (2.8)	2.2 (2.7)	2.5 (2.9)	2.0 (2.6)	2.4 (2.8)	2.2 (2.8)
**Physically active (**at least 2 x week at baseline) [4], n (%)	882 (70.8%)	841 (71.4%)	41 (60.3%)	805 (70.9%)	77 (69.4%)	685 (72.2%)	197 (66.3%)	604 (72.4%)	278 (67.5%)	528 (72.4%)	354 (68.5%)
**Smoking** at baseline, n(%)	114 (9.4%)	103 (9%)	11 15.9%)	100 (9.1%)	14 (13.2%)	70 (7.6%)	44 (15.3%)***	67 (8.3%)	47 (11.8%)*	53 (7.5%)	61 (12.2%)**
**Alcohol use** less than weekly at baseline[4], n (%)	695 (55.6%)	654 (55.4%)	41 (57.7%)	626 (55.1%)	69 (60.0%)	518 (54.5%)	177 (58.8%)	454 (54.4%)	241 (57.9%)	400 (54.8%)	295 (56.6%)
**Binge drinking** less than monthly at baseline[4], n (%)	1023 (86.8%)	970 (87.0%)	53 (84.1%)	936 (87%)	87 (85.3%)	789 (87.4%)	234 (85.1%)	694 (87.6%)	329 (85.2%)	610 (87.5%)	413 (85.9%)
**Fish** at baseline (times per week), mean (SD)	1.7 (1.1)	1.7 (1.1)	1.7 (1.3)	1.7 (1.1)	1.8 (1.3)	1.7 (1.2)	1.7 (1.0)	1.7 (1.1)	1.7 (1.1)	1.7 (1.1)	1.7 (1.1)
**Fruits and berries** at least daily at baseline[4], n(%)	812 (64.6%)	769 (64.8%)	43 (60.6%)	733 (64.2%)	79 (68.7%)	615 (64.4%)	197 (65.2%)	536 (63.8%)	276 (66.2%)	487 (66.4%)	325 (62.0%)
**Vegetables and roots** at least daily at baseline[4], n(%)	775 (61.7%)	735 (62.0%)	40 (56.3%)	702 (61.5%)	73 (63.5%)	603 (63.2%)	172 (57.0%)	531 (63.3%)	244 (58.5%)	466 (63.7%)	309 (59%)

**Good self-evaluated health**[3], n(%)	750 (59.9%)	713 (60.3%)	37 (52.9%)	693 (60.9%)	57 (50%)*	589 (61.9%)	161 (53.5%)**	521 (62.2%)	229 (55.2%)*	470 (64.4%)	280 (53.6%)**
**Good self-evaluated memory**[3]**,** n(%)	596 (47.4%)	564 (47.6%)	32 (45.1%)	550 (48.2%)	46 (40.0%)	468 (49.0%)	128 (42.4%)*	413 (49.2%)	183 (43.9%)	374 (51.0%)	222 (42.4%)**

Ps, participants; non-ps, non-participants. It was possible for non-participants to return to the study during later visits.

Changes reflect differences in participation in different time points. Statistically significant differences between participants and those who dropped out at different time points are presented in superscripts (*p<0.05, **p<0.01, ***p<0.001). [1]Questionnaire in summer 2020, follow-up time on average 10 years.

[2]Hypertension, diabetes, and depression were self-reported by participants at baseline.

[3] Good or very good

[4] Categories of the original question have been combined to dichotomise variable only for the purpose of this table (analyses conducted with the original categories).
