# Supplementary material for: Changes in older persons’ lifestyle and perceived health over time and during the COVID-19 pandemic: findings from the extended follow-up of the FINGER randomized controlled trial from 2009 to 2020
Source: BMC Geriatr. 2025 May 3;25:308. doi: 10.1186/s12877-025-05979-6 (PMC12048948; doi:10.1186/s12877-025-05979-6)
Supplement: Supplementary file 3 — Additional file 3. Pandemic related changes [file 12877_2025_5979_MOESM3_ESM.docx]

**Supplementary table 3.** Changes in lifestyles before and during the pandemic in the different groups, and differences between the groups.

|  | **Intervention** | | | **Control** | | | **Difference between groups (intervention-control)** | | |
| --- | --- | --- | --- | --- | --- | --- | --- | --- | --- |
|  | Contrast | SE | p-value | Contrast | SE | p-value | Contrast | SE | p-value |
| **Physical activity^1^** |  |  |  |  |  |  |  |  |  |
| 7y-5y | -0.167 | 0.099 | 0.090 | 0.019 | 0.096 | 0.847 | -0.185 | 0.138 | 0.179 |
| Pandemic-7y | 0.693 | 0.114 | <0.001 | 0.963 | 0.121 | <0.001 | -0.270 | 0.132 | 0.041 |
| **Cognitive activity^2^** |  |  |  |  |  |  |  |  |  |
| 7y-5y | -0.138 | 0.213 | 0.517 | -0.262 | 0.211 | 0.214 | 0.124 | 0.300 | 0.680 |
| Pandemic-7y | 0.083 | 0.227 | 0.714 | 0.479 | 0.226 | 0.034 | -0.395 | 0.321 | 0.217 |
| **Social activity^2^** |  |  |  |  |  |  |  |  |  |
| 7y-5y | 0.022 | 0.131 | 0.865 | -0.385 | 0.129 | 0.003 | 0.407 | 0.184 | 0.027 |
| Pandemic-7y | -0.774 | 0.139 | <0.001 | -0.495 | 0.139 | <0.001 | -0.279 | 0.196 | 0.155 |
| **Smoking^1^** |  |  |  |  |  |  |  |  |  |
| 7y-5y | -0.239 | 0.160 | 0.137 | 0.042 | 0.182 | 0.819 | -0.280 | 0.242 | 0.247 |
| Pandemic-7y | -0.329 | 0.205 | 0.108 | -0.118 | 0.127 | 0.355 | -0.211 | 0.242 | 0.382 |
| **Alcohol use^1^** |  |  |  |  |  |  |  |  |  |
| 7y-5y | -0.087 | 0.102 | 0.394 | -0.153 | 0.105 | 0.145 | 0.067 | 0.143 | 0.641 |
| Pandemic-7y | -0.435 | 0.136 | 0.001 | -0.413 | 0.133 | 0.002 | -0.022 | 0.142 | 0.876 |
| **Binge drinking^1^** |  |  |  |  |  |  |  |  |  |
| 7y-5y | -0.083 | 0.054 | 0.123 | -0.071 | 0.049 | 0.143 | 0.011 | 0.059 | 0.847 |
| Pandemic-7y | 0.083 | 0.053 | 0.115 | 0.130 | 0.064 | 0.043 | -0.047 | 0.062 | 0.451 |
| **Self-evaluated health^1^** |  |  |  |  |  |  |  |  |  |
| 7y-5y | -0.084 | 0.042 | 0.045 | -0.101 | 0.043 | 0.018 | 0.017 | 0.057 | 0.764 |
| Pandemic-7y | -0.120 | 0.048 | 0.012 | -0.106 | 0.047 | 0.024 | -0.015 | 0.059 | 0.801 |
| **Self-evaluated memory^1^** |  |  |  |  |  |  |  |  |  |
| 7y-5y | -0.041 | 0.039 | 0.289 | -0.021 | 0.039 | 0.597 | -0.021 | 0.055 | 0.704 |
| Pandemic-7y | -0.077 | 0.044 | 0.081 | -0.046 | 0.041 | 0.261 | -0.030 | 0.056 | 0.589 |

Changes in lifestyle variables in the intervention and control groups, and differences between the groups during the latest 2-year period before the pandemic (5 to 7 years) and the period up to pandemic (approximately 2 years; 7 to pandemic). Contrasts with their standard errors and p-values are presented; positive values refer to increase and negative values to decrease. ^1^Generalized estimating equations (GEE) used in analysis. ^2^Linear mixed-effects regression model used in analysis.
